# Adjuvant immunotherapy in early-stage resectable non–small cell lung cancer: A new milestone

**DOI:** 10.3389/fonc.2023.1063183

**Published:** 2023-01-26

**Authors:** Wen-Fang Tang, Hong-Yu Ye, Xuan Tang, Jian-Wei Su, Kang-Mei Xu, Wen-Zhao Zhong, Yi Liang

**Affiliations:** ^1^ Department of Cardiothoracic Surgery, Zhongshan City People’s Hospital, Zhongshan, Guangdong, China; ^2^ Guangdong Lung Cancer Institute, Guangdong Provincial People's Hospital (Guangdong Academy of Medical Sciences), Southern Medical University, Guangzhou, Guangdong, China

**Keywords:** resectable non-small cell lung cancer, adjuvant immunotherapy, immune checkpoint inhibition, immune suppression environment, chemotherapy

## Abstract

Currently, chemotherapy is the standard adjuvant treatment for early-stage non-small cell lung cancer (NSCLC). However, adjuvant cisplatin-based chemotherapy after surgery has been shown to improve 5-year survival rates by only 4−5%. Immunotherapy using immune checkpoint inhibitors (ICIs) has revolutionized the treatment of advanced NSCLC, there is a growing interest in the role of immunotherapy in early-stage NSCLC. Here, we summarize the rationale for adjuvant immunotherapy, including the postoperative immunosuppressive environment and immunological effects of platinum chemotherapy. Many ongoing clinical trials and the related progress in adjuvant immunotherapy in early-stage resectable NSCLC are discussed. Furthermore, we highlight several unresolved challenges, including markers predictive of treatment benefit, the efficacy of treatment for some oncogene-addicted tumors, the optimal combination therapy, the duration of adjuvant immunotherapy, and optimal selection between neoadjuvant and adjuvant immunotherapy. Early findings in some clinical trials are promising, and updated overall survival results will be useful for validating the current role of adjuvant immunotherapy, particularly in the context of perioperative strategy.

## 1 Introduction

Lung cancer continues to be the leading cause of cancer mortality globally (1). Non-small cell lung cancer (NSCLC) represents 80-85% of newly diagnosed lung cancer cases (2, 3). Overall, approximately 50% of NSCLC patients present with localized (stages I and II) or locally advanced (stage III) disease (4). Even after curative surgery, 5-year survival rates decrease from 90% to 24% with increasing stage due to recurrence and metastasis (5), which indicates that micrometastases are present in some patients at surgical resection. Thus, improving the cure rate of early-stage NSCLC is currently one of the major challenges.

Adjuvant therapy plays an important role in eliminating micrometastases and preventing recurrence. Osimertinib (6), icotinib (7), gefitinib (8) and erlotinib (9) as adjuvant targeted treatments showed better disease-free survival (DFS) benefits than chemotherapy (30.8-47.0 vs. 19.8-22.1 months) for patients with NSCLC with epidermal growth factor receptor (EGFR) mutations. In ADAURA (6), at 24 months, 90% of the patients with stage II to IIIA NSCLC disease in the osimertinib group were disease-free, but only 44% of those in the placebo group (hazard ratio (HR)=0.17; P<0.001). However, for most patients with early-stage NSCLC who have EGFR wild-type tumors, standard adjuvant chemotherapy (10, 11) resulted in only a 4-5% improvement in the 5-year survival rate compared with that of surgery alone (12, 13).

Immunotherapy based on immune checkpoint inhibitor (ICI) has revolutionized the treatment of unresectable locally advanced or metastatic NSCLC and has gradually moved from being a second-line treatment to a first-line treatment option (14–16). Recent studies have shown that ICI monotherapy and ICI in combination with chemotherapy improved survival in advanced NSCLC, with some trials showing an association among programmed death-ligand 1 (PD-L1) expression and treatment benefit (17–23). Based on the success of ICI in metastatic disease, there is a growing interest in expanding its use in patients with early-stage NSCLC. In this review, we will focus on the current status of adjuvant immunotherapy in early-stage resectable NSCLC.

## 2 Rationale for adjuvant therapy

### 2.1 Postoperative immunosuppressive environment

**Figure 1:**
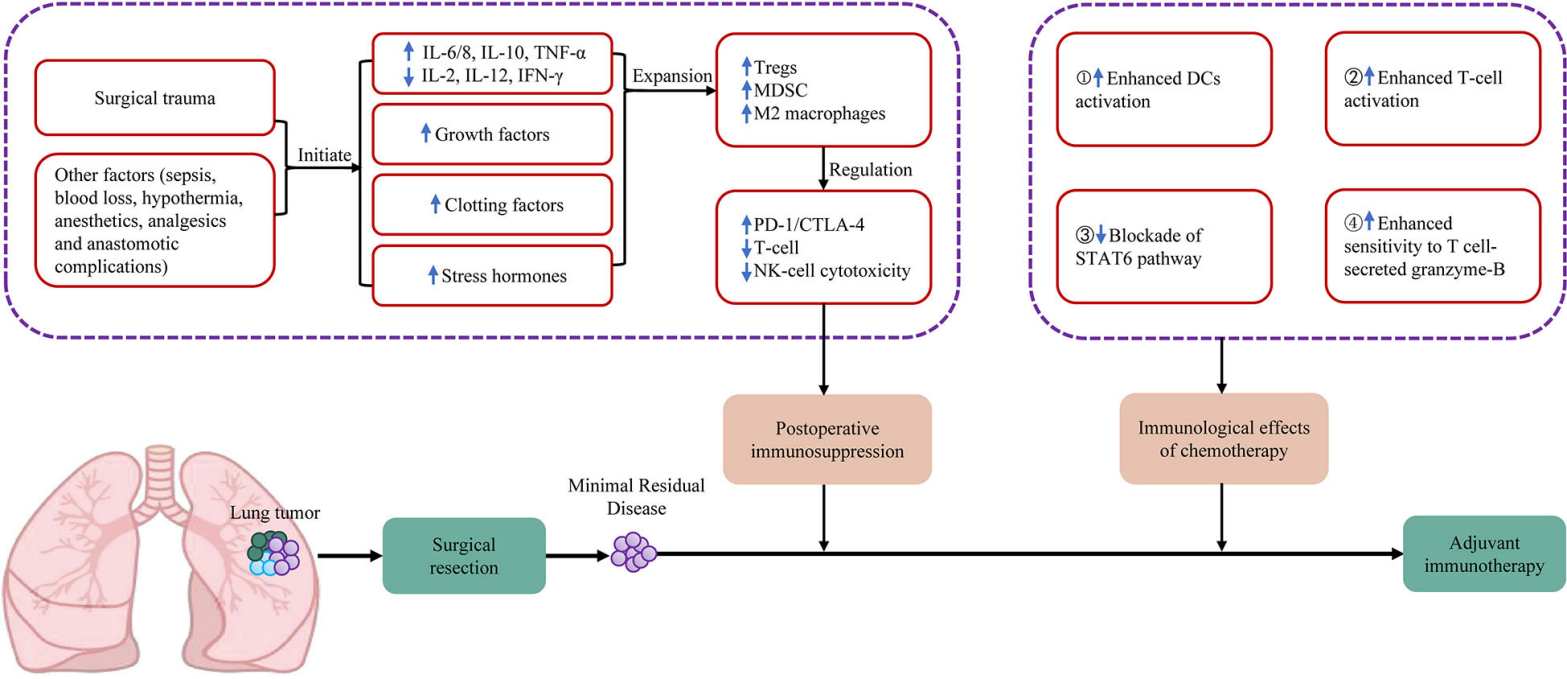
Summary of mechanisms of postoperative immunosuppression and immunological effects of chemotherapy on the tumour microenvironment. CTLA-4, cytotoxic T-lymphocyte-associated protein 4; DCs, dendritic cells; IFN, interferon; IL, interleukin; MDSC, myeloid derived suppressor cells; NK cell, natural killer cell; PD-1, programmed cell death protein 1; STAT6, signal transducer and activator of transcription 6; TNF, tumor necrosis factor; Tregs, regulatory T cells.

Cancer surgery-induced immune dysfunction provides a theoretical basis for the use of ICI as adjuvant therapy (24). Surgical resection induces inflammatory responses and metabolic events (25). As a result, altered cytokine levels occur, and Th1 cytokines (IL-2, IL-12 and IFN-γ) are suppressed, but Th2 immunity is increased (IL-10, IL-6/8, TNF-α) (26–28), which is characterized by the release of clotting factors, growth factors (such as PDGF, VEGF, TGF-β) and stress hormones (prostaglandins, catecholamines) (28). These events are key regulators of wound healing and pain control, and they can also lead to the rapid expansion of myeloid-derived suppressor cells (MDSCs), M2 macrophages and T regulatory cells (Tregs) (24). Cellular immune suppression leads to the expression of programmed cell death protein 1 (PD-1)/cytotoxic T lymphocyte associated protein 4 (CTLA-4) (29), T-cell dysfunction (30–32) and NK-cell functional impairment (33, 34), overall resulting in postoperative immunosuppression in the patient (**Figure 1**). Alongside surgical trauma, other postoperative factors, such as hypothermia, blood loss and sepsis, contribute to immunosuppression (24). In contrast, ICI can block the binding of PD-1 and PD-L1, upregulate the growth and proliferation of T cells, enhance the recognition of tumor cells by T cells, and activate their attack and killing functions, thus realizing their antitumor effect.

### 2.2 Immunological effects of chemotherapy

The combination of chemotherapy with immunotherapy may have a synergistic effect and is more effective in removing postoperative minimal residual disease (3) (**Figure 1**). A normal immune response requires the ability of T cells to recognize nonself antigens (antigenicity) and innate immune cells to sense danger signals in the environment (adjuvanticity). These processes activate the innate immune system, promote dendritic cell maturation, and eventually activate effector T cells. The third function of the immune system is homeostatic feedback inhibition, which can curb the immune response in a timely manner to prevent immune-mediated self-damage after the clearance of pathogens. Cancer has developed numerous strategies to modulate the normal immune response (35). For example, a loss of tumor antigen expression or presentation contributes to tumor immune evasion. Danger signals released by dying cancer cells promote cancer inflammation. The infiltration of immunosuppressive cells, the release of immunosuppressive molecules and the stimulation of feedback inhibition by various checkpoint molecules eventually activate the formation of an immunosuppressive microenvironment. Tumor cells disrupt the discriminatory functions of the immune response to evade elimination through the following mechanisms: (I) reduced class I MHC expression or genetic loss of B2-microglobulin to prevent cell surface presentation of T cells recognizing tumor-associated antigens (TAAs); (II) tumor cells may inhibit cell-intrinsic activation of pattern recognition receptor (PRR) signaling through the genetic loss or silencing of pathways such as cGAS/STING; and (III) the feedback mechanisms that suppress normal immune responses can be exploited by tumor cells.

Chemotherapy can exert immunomodulatory effects not only by affecting tumor cells but also by increasing immunogenicity and increasing T-cell infiltration (36, 37). Cancer cells with innate or experimentally enforced defects cause immunogenic cell death (ICD). The adaptability of chemotherapy is enhanced by the induction of ICD. ICD can be induced by different stressors, including (I) pathogens; (II) physical modalities, encompassing high hydrostatic pressure, severe heat shock, various forms of ionizing radiation, etc.; (III) chemotherapeutics such as anthracyclines and DNA-damaging agents; and (IV) targeted anticancer agents such as the epidermal growth factor receptor-specific monoclonal antibody cetuximab. The mechanism of chemotherapy-driven ICD is as follows (38): In response to chemotherapeutic agents, malignant cell surfaces express calreticulin (CALR) and other endoplasmic reticulum (ER) chaperones and secrete ATP, resulting in a type I interferon (IFN) response that stimulates the production of CXC-chemokine ligand 10 (CXCL10) and releases high-mobility group Box 1 (HMGB1) and annexin A1 (ANXA1). These processes facilitate the uptake of cell corpses and fragments by antigen-presenting cells, including dendritic cells (DCs), and lead to the activation of the adaptive immune response involving T cells.

In advanced NSCLC, the combination of an immune checkpoint inhibitor and either pemetrexed or paclitaxel with cisplatin- or carboplatin-based chemotherapy as first-line therapy obtains significant therapeutic effects. At present, among adjuvant ICIs in combination with chemotherapy, the chemotherapy regimen is mostly based on platinum-based compound. The immunogenic effects of platinum compounds include the following four aspects (3, 39) (**Figure 1**): (I) the induction of ICD; (II) the downregulation of PD-L1 and PD-L2 on DCs and the improvement in their T-cell activation potential; (III) the inactivation of the STAT pathway, thereby decreasing the expression of PD-L2 on cancer cells; and (IV) the upregulation of the M6P receptor on tumor cells, leading to tumor cell killing by the granzyme-B of activated T cells.

## 3 Clinical trials of adjuvant immunotherapy in patients with resectable NSCLC

**Figure 2:**
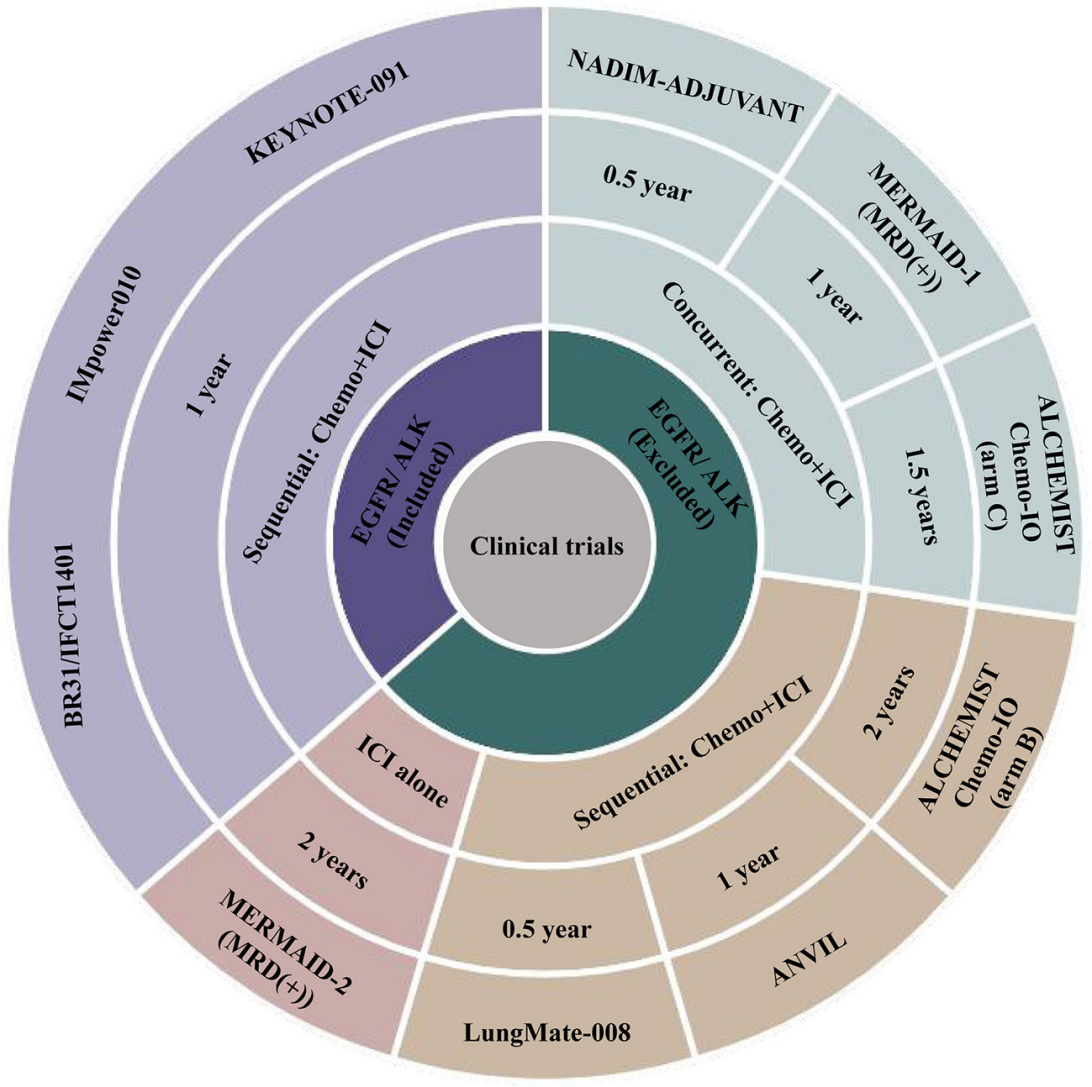
Summary of ongoing adjuvant immunotherapy clinical trials. ALK, anaplastic lymphoma kinase; Chemo, chemotherapy; EGFR, epidermal growth factor receptor; ICI, immune checkpoint inhibitor.

Currently, most of the ongoing clinical trials of adjuvant immunotherapy for resectable NSCLC involve chemotherapy with concurrent or subsequent immunotherapy (**Figure 2**) in patients with stage IB-IIIA NSCLC and resectable stage IIIB (T>5 cm and N2) NSCLC. Approximately half of these trials did not exclude patients with EGFR mutations- or anaplastic lymphoma kinase (ALK) rearrangements-positive NSCLC. Furthermore, the MERMAID-1 and MERMAID-2 studies used the presence of minimal residual disease (MRD) as the inclusion criterion. The efficacy and safety of adjuvant ICI monotherapy or ICI in combination with chemotherapy in patients with completely resected stage II/III NSCLC are being investigated to assess the benefits of adjuvant therapy in patients with MRD-positive status. The results of these trials will provide new insights for clinical applications. The details of each clinical trial are as follows (**Table 1**).

**Table 1:** Ongoing clinical trials that included adjuvant immunotherapy in resectable non-small cell lung cancer.

Trial	NCT Number	Sponsor	Start date	Phase	Stage	Participants (*n*)	EGFR mutation/ALK rearrangement	Intervention following surgery	Primary endpoint	Completion date
BR31/IFCT1401	NCT02273375	Canadian Cancer Trials Group	2014-10-9	3	IB-IIIAAJCC 7th	1415	Included	**Arm A:** (optional chemotherapy and RT if N2) Durvalumab (1 year); **arm B:** (optional chemotherapy and RT if N2) placebo (1 year)	DFS	2024-1-31
IMpower010	NCT02486718	Hoffmann-La Roche	2015-10-31	3	IB-IIIA AJCC 7^th^	1280	Included	**Arm A:** cisplatin-based chemotherapy (4 cycles, Q3W) then atezolizumab (16 cycles, Q3W); **arm B:** cisplatin-based chemotherapy (4 cycles, Q3W) then best supportive care	DFS	2027-12-17
PEARLS/KEYNOTE-091	NCT02504372	Merck Sharp &Dohme LLC	2015-11-6	3	IB-IIIA AJCC 7^th^	1177	Included	**Arm A:** (optional chemotherapy) pembrolizumab (1 year, Q3W); **arm B:** (optional chemotherapy) placbo (1 year, Q3W))	DFS	2024-2-2
ANVIL	NCT02595944	National CancerInstitute (NCI)	2016-5-6	3	IB-IIIA AJCC 7^th^	903	Excluded	**Arm A:** (optional chemotherapy and RT) nivolumab (1 year, Q4W); **arm B**: (optional chemotherapy and RT) observation (1 year)	DFS, OS	2024-7-1
ALCHEMIST Chemo-IO	NCT04267848	National CancerInstitute (NCI)	2020-6-3	3	IIA–IIIBAJCC 8^th^	1210	Excluded (applicable to non-squamous NSCLC)	**Arm A:** platinum doublet (4 cycles, Q3W) then observation; **arm B:** platinum doublet (4 cycles, Q3W) then pembrolizumab 17 cycles (Q3W) or 16 cycles (Q6W, after 10/14/2020); **arm C:** platinum doublet plus pembrolizumab (4 cycles, Q3W) , then pembrolizumab 13 cycles (Q3W) or 12 cycles (Q6W, after 10/14/2020)	DFS	2024-12-15
MERMAID-1	NCT04385368	AstraZeneca	2020-7-17	3	II-III AJCC 8th	332	Excluded	MRD+ patients; **Arm A**: Durvalumab plus SoC chemotherapy 4 cycles (Q3W), followed by 12 cycles (Q4W); **arm B**: placebo plus SoC chemotherapy 4 cycles (Q3W), followed by 12 cycles (Q4W)	DFS	2026-9-30
MERMAID-2	NCT04642469	AstraZeneca	2020-11-30	3	II-IIIAJCC 8^th^	284	Excluded	**MRD regular monitor:** MRD+ patients receive durvalumab versus placebo (2years, Q4W)	DFS	2027-10-29
NADIMADJUVANT	NCT04564157	Fundación GECP	2021-1-13	3	IB-IIIAAJCC 8^th^	210	Excluded	**Arm A**: Paclitaxel+carboplatin+nivolumab (4 cycles, Q3W) then nivolumab (6 cycles, Q4W); **Arm B**: Paclitaxel+carboplatin (4 cycles, Q3W) then observation	DFS	2028-4-1
LungMate-008	NCT04772287	Shanghai Pulmonary Hospital	2021-3-31	3	II-IIIBAJCC 8^th^	341	Excluded	**Arm A:** platinum doublet (4 cycles, Q3W) then toripalimab (4 cycles, Q3W); **arm B:** platinum doublet (4 cycles, Q3W) then placebo (4 cycles, Q3W)	DFS	2027-12-30

AJCC, American Joint Committee on Cancer; ALK, anaplastic lymphoma kinase; DFS, disease-free survival; EGFR, epidermal growth factor receptor; MRD, minimal residual disease; NCT, national clinical trial; OS, overall survival; RT, radiotherapy; SoC, standard of care.

### 3.1 BR31/IFCT1401 (NCT02273375)

The BR31/IFCT1401 trial is a double-blind phase III trial involving patients with completely resected NSCLC (stage IB–IIIA, American Joint Committee on Cancer (AJCC) 7th tumor node metastasis (TNM) stage), initiated in October 2014 with a completion date of January 2024 (40). A total of 1415 participants are estimated to be enrolled, and patients with NSCLC with EGFR mutations or ALK rearrangements are not excluded. After receiving optional adjuvant chemotherapy, participants are randomized to receive either durvalumab or placebo for a year. The primary outcome measures include disease-free survival (DFS) for patients with NSCLC with PD-L1 expression TC ≥25% and patients with NSCLC without EGFR mutations or ALK rearrangements.

### 3.2 IMpower010 (NCT02486718)

The IMpower010 trial was launched in October 2015 and has an estimated completion date of December 2027. It is an open-label phase III study with a sample size of 1280 patients with resected NSCLC (stage IB–IIIA, AJCC 7th TNM stage) from 227 sites in 22 countries (41). This trial also includes patients with NSCLC tumors with EGFR mutations or ALK rearrangements. After completing up to four cycles (q3w) of adjuvant cisplatin-based chemotherapy, eligible participants are randomized to receive atezolizumab for 16 cycles (q3w) or best supportive care. The primary outcome is DFS, for which the results have been published previously (41).

### 3.3 KEYNOTE-091 (NCT02504372)

This phase III trial was initiated in November 2015 and is scheduled to be completed in February 2024. A total of 1177 patients with resected NSCLC (stage IB–IIIA, AJCC 7th TNM stage) (42) are enrolled in the study. After completing optional adjuvant chemotherapy, participants are randomized to receive adjuvant pembrolizumab or placebo every 3 weeks for one year. Furthermore, NSCLC patients with EGFR/ALK (+) tumors were not excluded. The primary endpoint of the study is DFS.

### 3.4 ANVIL (NCT02595944)

A total of 903 participants with resected NSCLC (stage IB-IIIA, AJCC 7th TNM stage) are enrolled in this trial (43), which was launched in May 2016 and is scheduled to be completed in July 2024. Patients with EGFR/ALK wild-type tumors were included in this study. After optional chemotherapy and radiotherapy, participants are randomized to receive adjuvant nivolumab every 4 weeks or observation for one year. The primary outcomes are DFS and OS. The secondary outcome measure is the incidence of toxicity grade.

### 3.5 ALCHEMIST Chemo-IO (NCT04267848)

The ALCHEMIST Chemo-IO Study was launched by the National Cancer Center in June 2020 with an estimated completion date of December 2024 (44). It is estimated to enroll 1210 stage IIA–IIIB (AJCC 8th TNM stage) participants with NSCLC without EGFR mutations or ALK rearrangements (applicable only to patients with nonsquamous tumors). Patients will be randomized to one of 3 treatment arms: chemotherapy-immunotherapy with pembrolizumab during (4 cycles, q3w) and after (12 cycles, q6w) chemotherapy, sequential chemotherapy (4 cycles, q3w) followed by pembrolizumab (16 cycles, q6w), or chemotherapy alone (4 cycles, q3w). The primary endpoint is DFS.

### 3.6 MERMAID-1 (NCT04385368)

As a phase III, multicenter, double-blind study initiated in July 2020, MERMAID-1 will recruit approximately 332 MRD-positive patients with completely resected stage II–III NSCLC (AJCC 8th TNM stage) without EGFR mutations or ALK rearrangements (45). Patients will be randomized to receive adjuvant durvalumab plus standard of care (SOC) chemotherapy or placebo plus SOC chemotherapy (4 cycles (q3w) followed by 12 cycles (q4w)). The primary outcome measure is DFS in the MRD-positive analysis set, and the estimated study completion date is September 2026.

### 3.7 MERMAID-2 (NCT04642469)

The MERMAID-2 trial is a phase III, multicenter double-blind study launched in November 2020 (46) with an estimated completion date of October 2027. A total of 284 participants are estimated to be enrolled in the study. After complete resection, patients with stage II–III NSCLC (AJCC 8th TNM stage) without EGFR mutations or ALK rearrangements will be regularly monitored for the presence of MRD *via* the analysis of circulating tumor DNA levels in plasma samples. Patients who become MRD+ during the surveillance period and have no disease recurrence visible on imaging will be randomized 1:1 to receive adjuvant durvalumab or placebo every 4 weeks for up to two years or until investigator-assessed disease recurrence. The primary endpoint is DFS in patients with PD-L1 tumor cell expression (TC) ≥ 1%.

### 3.8 NADIM-ADJUVANT (NCT04564157)

The NADIM-ADJUVANT trial is an open-label, randomized, two-arm, phase III, multicenter clinical trial (47) that was launched in January 2021 with an expected completion date of April 2028. A total of 210 participants (stage IB-IIIA NSCLC without EGFR mutations or ALK rearrangements) are estimated to be recruited. Patients will be randomized to one of 2 treatment arms: chemotherapy-immunotherapy with nivolumab during (4 cycles, q3w) and after (6 cycles, q4w) chemotherapy or chemotherapy alone (4 cycles, q3w). The primary endpoint is DFS. This trial was launched by January 2021 and will be completed in April 2028.

### 3.9 LungMate-008 (NCT04772287)

This study is a randomized, double-blind, controlled phase III trial in regard to adjuvant toripalimab versus placebo combined with chemotherapy for EGFR/ALK mutation-negative stage II-IIIB (N2) (AJCC 8th TNM stage) NSCLC (48). After adjuvant chemotherapy (4 cycles, q3w), 341 patients were randomized to receive adjuvant toripalimab or placebo for 4 cycles (q3w). The primary endpoint is DFS, and this trial will be completed in December 2027.

## 4 Available results from adjuvant immunotherapy clinical trials

Initially, because many patients with resected NSCLC are often unable to tolerate standard adjuvant cisplatin-based chemotherapy, which was proven to result in a modest 4–5% improvement in survival (12, 13), scientists began to focus on therapeutic cancer vaccines with minimal toxicity. Melanoma-associated antigen (MAGE)-A3 is a tumor-specific antigen expressed in 30–50% of NSCLC tumors (49). Kruit et al. found that immunotherapy with recombinant MAGE-A3 protein has antitumor efficacy in patients with metastatic melanoma (50). In a preliminary phase II trial (51), the MAGE-A3 vaccine exhibited a survival benefit in patients with completely resected MAGE-A3-positive stage IB-II NSCLC. In the ensuing randomized, double-blind, phase III trial (MAGRIT trial) (52), a total of 2272 patients were randomly assigned (2:1) to receive MAGE-A3 immunotherapy or placebo. Unfortunately, the MAGE-A3 immunotherapeutic did not lead to better DFS than the placebo (60.5 vs. 57.9 months, HR=1.02). These results may be attributed to the low level of the CD8+ T-cell response (53) and the possibility that MAGE-A3-mediated lymphocytes may be inactivated and thus fail to kill tumor cells in the postoperative immunosuppressive tumor microenvironment (3).

The efficacy and safety of canakinumab, an interleukin-1β blocker, was evaluated in a phase III clinical trial (CANOPY-A study, NCT03447769). A total of 1382 patients with completely resected NSCLC with or without EGFR mutations or ALK rearrangements (stage IIA–IIIA, IIIB with N2 disease only, AJCC 8th TNM stage) were enrolled in this trial (54), which began in March 2018. Patients who received adjuvant cisplatin-based chemotherapy (≥2 cycles) were allowed to join the study. Then, they were randomized to adjuvant canakinumab or placebo for 18 cycles (q3w). The primary endpoint was DFS, as assessed by a local investigator. Unfortunately, on August 15, 2022, Novartis announced that this study did not reach the DFS primary endpoint. The results were presented at the 2022 European Society for Medical Oncology Annual Meeting (ESMO 2022) (55). Compared with placebo, canakinumab did not significantly improve DFS (35.0 vs. 29.7 months; HR=0.94; 95% confidence interval (CI): 0.78-1.14).

In contrast, ICI immunotherapy exhibited exciting results in patients with early resectable NSCLC. The IMpower010 trial was the first published phase III clinical study to demonstrate the efficacy of ICI in the adjuvant immunotherapy of NSCLC (41). A total of 1208 patients were enrolled, and 1005 patients were finally randomized (1:1) to receive adjuvant atezolizumab (n=507) or best supportive care (n=498). After a median follow-up of 32.2 months, the primary endpoint, DFS, was reached. In patients with stage II–IIIA NSCLC whose tumors expressed PD-L1 TC ≥1%, 35% (88/248) of patients receiving atezolizumab and 46% (105/228) of patients receiving best supportive care experienced disease progression, indicating that adjuvant atezolizumab could reduce the risk of recurrence by 34% (HR=0.66; 95% CI: 0.50–0.88). Therefore, in October 2021 (56), the United States Food and Drug Administration (FDA) approved atezolizumab monotherapy as adjuvant therapy in patients with PD-L1-positive (TC ≥1%) stage II-IIIA NSCLC after surgical resection and platinum-based chemotherapy.

**Figure 3:**
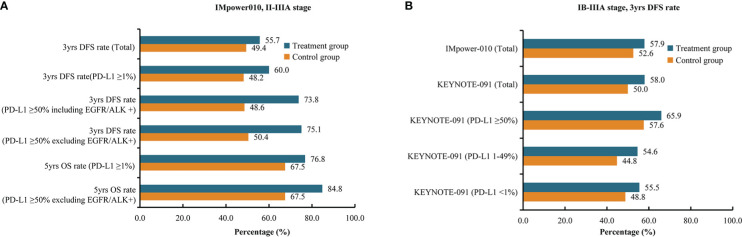
Summary of survival rates of clinical trials. **(A)** 3-year disease-free survival rates and 5-year overall survival rates of patients with stage II-IIIA non–small cell lung cancer in the IMpower010 trial. **(B)** 3-year disease-free survival rates of patients with stage IB-IIIA non-small cell lung cancer in the IMpower010 and KEYNOTE-091 trials. ALK, anaplastic lymphoma kinase; Chemo, chemotherapy; DFS, disease-free survival; EGFR, epidermal growth factor receptor; OS, overall survival; PD-L1, programmed death-ligand 1.

At the 2022 European Lung Cancer Congress (ELCC 2022) (57), IMpower010 trial investigators updated the survival data of the PD-L1 TC ≥50% stage II–IIIA population subgroup. The atezolizumab group showed a better DFS benefit than the best supportive care group among the patients with PD-L1 TC ≥50% stage II–IIIA NSCLC, with or without EGFR mutations or ALK rearrangements. When patients with EGFR mutations- or ALK rearrangements-positive tumors were included, the 3-year DFS rates in the atezolizumab treatment group and the control group were 73.8% and 48.6%, respectively (HR=0.43; 95% CI: 0.27-0.68). After excluding patients with EGFR mutations- or ALK rearrangements-positive tumors, the 3-year DFS rates in the atezolizumab group and control group were 75.1% and 50.4%, respectively (HR=0.43; 95% CI: 0.26-0.71) (**Figure 3A**). Compared with best supportive care, adjuvant atezolizumab treatment was associated with a lower risk of recurrence (recurrence rate: 22% vs. 44%), a longer median recurrence time (18.1 vs. 10.1 months) and a lower rate of distant metastasis, including metastasis to the central nervous system (9% vs. 26%). Furthermore, the OS interim analysis results of the IMpower010 trial were presented at the 2022 World Conference on Lung Cancer (WCLC 2022) (58). After a median follow-up of 45.3 months, the atezolizumab group showed a better OS trend than the best supportive care group among patients with PD-L1 TC ≥1% with stage II-IIIA NSCLC (5-year OS rate: 76.8% vs. 67.5%). For patients with PD-L1 TC ≥50% stage II-IIIA NSCLC without EGFR mutations or ALK rearrangements, the 5-year OS rates in the atezolizumab group and control group were 84.8% and 67.5%, respectively (HR=0.42, 95% CI: 0.23-0.78) (**Figure 3A**).

For the KEYNOTE-091 trial (59), the results from interim analysis showed that DFS was significantly improved in stage IB-IIIA NSCLC patients receiving pembrolizumab compared with those receiving placebo, regardless of PD-L1 expression (53.6 months vs. 42.0 months; HR=0.76; 95% CI: 0.63-0.91). The 3-year DFS rates were 58.0% and 50.0%, respectively, in the total population (**Figure 3B**). However, for the PD-L1-positive (TC ≥50%) population, pembrolizumab did not improve DFS (3-year DFS rates: 66.0% vs. 58.0%), and the survival curves of the two groups crossed at 18 months (HR=0.82; 95% CI: 0.57-1.18). At the ESMO Congress in September 2022 (60), the investigators updated the DFS results according to PD-L1 expression. For the patients with PD-L1 TC ≥50%, the 3-year DFS rates in pembrolizumab and placebo arm were 65.9% and 57.6%, respectively (HR=0.82; 95% CI: 0.57-1.18). For PD-L1 TC 1-49% subgroup, the 3-year DFS rates in pembrolizumab and placebo arm were 54.6% and 44.8%, respectively (HR=0.67; 95% CI: 0.48-0.92). Moreover, the 3-year DFS rates in pembrolizumab and placebo arm were 55.5% and 48.8% in PD-L1 TC <1% subgroup (HR=0.78; 95% CI: 0.58-1.03) (**Figure 3B**).

## 5 Challenges and perspectives

Currently, the IMpower010 and KEYNOTE-091 trials have published preliminary results suggesting the effectiveness of ICI therapy in adjuvant immunotherapy after complete NSCLC resection. Furthermore, other clinical trials of adjuvant ICI immunotherapy, such as the BR31, ANVIL, ALCHEMIST Chemo-IO, MERMAID-1, MERMAID-2, NADIM-ADJUVANT and LungMate-008 studies, are ongoing. However, the following challenges remain.

### 5.1 Biomarkers predictive of benefit of adjuvant immunotherapy

As we all know, not all patients experience favorable survival benefits from immunotherapy. Therefore, it is important to identify patients most likely to benefit from adjuvant immunotherapy. The IMpower010 trial results suggested that PD-L1-positive (TC ≥1%) NSCLC patients can benefit from atezolizumab treatment. However, subgroup analysis showed that there was no significant difference in DFS between patients with PD-L1 expression of 1–49% in the atezolizumab group and the best supportive care group, indicating that the benefit of all groups was mainly derived from the group with high PD-L1 expression (TC ≥50%) (41). On the other hand, the KEYNOTE-091 study results were seemingly contradictory. The results showed that DFS was significantly improved in patients receiving pembrolizumab in the all-comer population, regardless of PD-L1 expression, but PD-L1 TC ≥50% NSCLC patients did not exhibit a survival benefit (59, 61). In conclusion, the predictive role of PD-L1 expression for adjuvant immunotherapy remains to be established. The ctDNA-based MRD was considered another potential biomarker for predicting the benefit of adjuvant therapy. In a cohort of 26 MRD-positive NSCLC patients, Liang et al. found that patients who received adjuvant treatment had significantly improved recurrence-free survival (RFS) compared with those who did not receive adjuvant treatment (HR=0.3; P=0.008) (62). Similarly, Zhang et al. revealed that patients with undetectable MRD might not benefit from adjuvant treatment in subgroup analyses (63). Furthermore, the MERMAID-1 and MERMAID-2 studies were launched in 2020 to evaluate the clinical application value of ctDNA-based MRD in predicting the benefits of adjuvant immunotherapy in NSCLC patients (45, 64). Finally, according to previous studies, blood-based TMb (65), tumor infiltrating lymphocytes (66) and specific gene mutations such as STK11, KEAP1 and TP53 (67) have been reported as biomarkers predictive of the benefits of ICI treatment. However, the value of those biomarkers for predicting the benefits of adjuvant immunotherapy are not clear, and more data are needed for validation.

### 5.2 Efficacy of adjuvant immunotherapy for some oncogene-addicted NSCLC

After the ADAURA study (6), osimertinib was approved as the standard adjuvant therapy for patients with stage IB-IIIA tumors with EGFR mutations (68). However, it is unclear whether there are patients with some oncogenes such as ALK rearrangements, KARS, STK11 or TP53 mutations that may benefit from immunotherapy in early stage (69). Furthermore, subsequent analysis from the ADJUVANT/CTONG1104 trial showed that patients with RB1-altered/EGFR-mutant tumors showed better survival with adjuvant chemotherapy than with adjuvant gefitinib (70); therefore, whether these patients can benefit from adjuvant immunotherapy remains to be determined.

### 5.3 Combined regimens of adjuvant immunotherapy

A combination of ICI and chemotherapy seemed to be associated with better survival than ICI monotherapy in patients with metastatic NSCLC (71, 72). Therefore, current phase III trials of adjuvant immunotherapy for early-stage NSCLC mainly assess the efficacy of sequential chemoimmunotherapy or concurrent chemoimmunotherapy, except for the MERMAID-2 trial, which evaluates the efficacy of durvalumab monotherapy for MRD+ NSCLC patients (**Figure 2**). However, whether sequential or concurrent immunochemotherapy provides a better benefit to patients remains unclear. Furthermore, there is no phase III trial comparing single-agent immunotherapy with chemoimmunotherapy. With increasing evidence showing the benefits of adjuvant immunotherapy, there is a need to compare different treatment regimens to optimize adjuvant therapy for early-stage NSCLC.

### 5.4 Duration of adjuvant immunotherapy

Currently, the recommended duration of atezolizumab as adjuvant immunotherapy is one year (73). However, as increasing evidence from clinical trials suggests, the duration of adjuvant immunotherapy may be as controversial as the duration of adjuvant targeted therapy, which is recommended to be 2-3 years (6–8). The duration of adjuvant immunotherapy in ongoing clinical trials ranges from 0.5 to 2 years (**Figure 2**), and we are looking forward to finding optimal patterns for the duration of adjuvant immunotherapy from these clinical trials.

### 5.5 Optimal selection between neoadjuvant and adjuvant immunotherapy

Neoadjuvant immunotherapy is considered another promising approach to improve survival in patients with resectable NSCLC and offers several advantages, such as the downstaging of the tumor, eliminating micrometastases earlier and improving tolerability. Related advances have been detailed in several reviews (64, 74, 75). CheckMate-816 was the first phase III neoadjuvant immunotherapy clinical trial for resectable NSCLC without EGFR mutations or ALK rearrangements (76). The median event-free survival was 31.6 months with nivolumab plus chemotherapy and 20.8 months with chemotherapy alone (P=0.005); the percentages of patients with a pathological complete response were 24.0% and 2.2%, respectively (P<0.001). Furthermore, Zhang et al. reported results for 40 patients with oncogene-mutant NSCLC treated with induction immunotherapy (77). The results suggested that the overall response rate was 62.5%, of which the major pathological response (MPR) rate was 37.5% and the pathological complete response (pCR) rate was 12.5%. The median disease-free survival for all patients with NSCLC with oncogenic mutations and EGFR mutations was 28.5 months. However, due to the overlap of NSCLC populations receiving neoadjuvant and adjuvant immunotherapy and the absence of survival benefit comparison between neoadjuvant and adjuvant immunotherapy, we need more data to help us to make the optimal selection.

## 6 Conclusions

The immunotherapies have changed the treatment landscape for patients with NSCLC in recent years. Current data suggested that lung resection followed by adjuvant immunotherapy is safe and feasible. Although the clinical trial data are still emerging, many factors remain to be determined, including predictors of response, the optimal combination and duration of adjuvant immunotherapy, and the integration with neoadjuvant immunotherapy. We need more studies to address these issues and to achieve precision medicine in NSCLC treatment.

## Author contributions

W-FT, XT wrote the manuscript and generated figures. HY, J-WS, W-ZZ, YL contributed to the concept and design and critically edited the manuscript. HY, W-ZZ, W-FT, YL, K-MX performed critical revision and editing of the scientific content. All authors contributed to the article and approved the submitted version
